# TEMPI综合征4例报告并文献复习

**DOI:** 10.3760/cma.j.issn.0253-2727.2023.08.013

**Published:** 2023-08

**Authors:** 婧钰 许, 明伟 傅, 军元 齐, 刚 安, 晓清 李

**Affiliations:** 1 中国医学科学院血液病医院（中国医学科学院血液学研究所），实验血液学国家重点实验室，国家血液系统疾病临床医学研究中心，细胞生态海河实验室，天津 300020 State Key Laboratory of Experimental Hematology, National Clinical Research Center for Blood Diseases, Haihe Laboratory of Cell Ecosystem, Institute of Hematology & Blood Diseases Hospital, Chinese Academy of Medical Sciences & Peking Union Medical College, Tianjin 300020, China; 2 深圳市第二人民医院（深圳大学第一附属医院），深圳市骨髓移植公共服务平台，深圳 518035 Shenzhen Second People's Hospital, the First Affiliated Hospital of Shenzhen University; Shengzhen Bone Marrow Transplantation Public Service Platform, Shenzhen 518035, China

TEMPI综合征的概念由2011年首次提出[1]，典型的临床表现包括：①毛细血管扩张（telangiectasias, T）；②促红细胞生成素（EPO）升高，HGB升高（elevated erythropoietin and erythrocytosis, E）；③单克隆丙种球蛋白病（monoclonal gammopathy, M）；④肾周积液（perinephric fluid, P）；⑤肺内分流（intrapulmonary shunting, I）。临床上将其归类为有皮肤意义的单克隆免疫球蛋白血症，在最新的《淋巴造血肿瘤WHO分类（第5版）》中，仍将TEMPI综合征归类为“浆细胞肿瘤伴副肿瘤综合征”[2]。目前国际上仅有20余例TEMPI综合征病例报道[3]，且患者常因出现红细胞增多及M蛋白而被误诊为真性红细胞增多症（PV）或意义未明单克隆丙种球蛋白血症（MGUS）。本文回顾性分析了中国医学科学院血液病医院和深圳市第二人民医院2017–2022年收治的4例TEMPI综合征患者的临床资料，并进行相关文献复习。

## 病例资料

例1，男，47岁，主因“发现HGB升高30个月余”入院。患者入院前2年因急性阑尾炎进行实验室检查示：RBC 6.87×10^12^/L，HGB 217 g/L，总蛋白（TP）95.6 g/L，EPO 41.3 mIU/ml（正常参考值：2.59～18.5 mIU/ml）；血清免疫固定电泳（IFE）：在γ区可见一条单克隆IgG κ成分；P190、P210、JAK2/V617F基因突变均阴性，当地医院考虑为PV，注射干扰素治疗1个月后疾病有所缓解，后间断行红细胞单采术（2014年1次，2015年2次，2016年2次），红细胞单采术后1个月HGB升高达190 g/L左右。入院查体：颜面潮红，胸背部可见毛细血管扩张。血常规：WBC 5.52×10^9^/L，RBC 7.53×10^12^/L，HGB 146 g/L，PLT 297×10^9^/L；生化常规：TP 108.6 g/L，球蛋白（GLO）62.2 g/L，血肌酐（Scr）102.7 µmol/L，EPO 253.38 mIU/ml；血管内皮生长因子（VEGF）355.7 pg/ml（正常参考值：0～142 pg/ml）；血清IFE：在γ区可见一条单克隆IgG κ成分；血清游离κ链16.6 mg/L，游离λ链11.80 mg/L，κ/λ＝1.4；血清M蛋白比例29.24％，血M蛋白定量31.75 g/L；血β_2_-微球蛋白（β_2_-MG）3.11 mg/L。尿蛋白电泳、IFE均阴性。血清细胞因子检测：巨噬细胞炎性蛋白（MIP-1α）18.15 pg/ml，单核细胞趋化蛋白-1（MCP-1）346.91 pg/ml。骨髓细胞学检查：幼稚浆细胞4.0％，成熟浆细胞12.0％。骨髓病理：骨髓增生极度活跃，红系比例增高，浆细胞散在分布（10％～20％），异常表达CD56，限制性表达Kappa，免疫组化：CD3（−），CD5（−），CD20（−），PAX5（−），CD56（+），CD38（+），Kappa（+），Lambda（−），CD138（+），CD10（−）。泌尿系B超：左肾体积增大，右肾体积正常，包膜规整，表面平滑，肾实质回声增强，皮质与髓质界限欠清。患者目前存在毛细血管扩张、EPO及HGB升高、M蛋白及异常浆细胞，考虑为TEMPI综合征，建议患者行以硼替佐米为基础的治疗，患者拒绝。近期电话随访，患者出现脑出血，仍有毛细血管扩张、乏力等。

例2，男，60岁，主因“反复皮肤毛细血管扩张10年，发现HGB升高1周”入院。患者5个月前查血常规示：WBC 6.13×10^9^/L，HGB 201 g/L，PLT 207×10^9^/L；生化常规：TP 77.4 g/L；EPO 36.3 mIU/ml；血IFE：IgG κ型M蛋白阳性；血清游离κ链24.3 mg/L，游离λ链13.0 mg/L，κ/λ 1.87；血清M蛋白比例8.8％，血M蛋白定量6.81 g/L。骨髓细胞学检查：红系以中晚幼红细胞为主，比例略高（28.5％），形态未见明显异常，浆细胞占5.5％。骨髓病理：骨髓增生活跃，粒红系增生活跃，未见明显异常病理细胞。免疫分型：可见CD38^+^CD138^+^异常浆细胞占有核细胞的0.15％，强表达CD38，表达CD138、CD56、CD200、cKappa，弱表达CD81，不表达CD19、CD20、CD45、CD117、cLambda，考虑为肿瘤性浆细胞。JAK2/V617F、CALR、MPL基因突变均阴性。右心超声造影提示肺内分流（大量）。腹部CT：右肾结节，考虑肿瘤性病变可能；右肾囊肿（部分为复杂囊肿）。患者存在毛细血管扩张、EPO及HGB升高、M蛋白、肺内分流，诊断为TEMPI综合征。间断予红细胞单采术2次及BD方案（硼替佐米，皮下注射2.3 mg/d，第1、8、15、22天；地塞米松20 mg/d，第1、2、8、9、15、16、22、23天）3个疗程。复查血常规示：WBC 9.49×10^9^/L，RBC 6.21×10^12^/L，HGB 171 g/L，PLT 244×10^9^/L。生化常规：TP 75.6 g/L，GLO 32.6 g/L，EPO 5.36 mIU/ml；β_2_-MG 2.79 mg/L；血IFE及M蛋白均阴性；血清游离κ链13.2 mg/L，游离λ链20.2 mg/L，κ/λ 为0.65。骨髓细胞学检查：三系增生骨髓象；骨髓及外周血成熟红细胞轻度堆积分布。骨髓病理：送检骨髓增生大致正常，粒系、红系、巨核系三系细胞增生，未见异常浆细胞明显增多。免疫分型：骨髓中正常浆细胞占有核细胞的0.20％，强表达CD138、CD38，表达CD19、CD27、CD81，不表达CD20、CD56、CD117、CD28、CD200，未见异常浆细胞。JAK2/V617F基因突变阴性。考虑治疗有效，建议患者继续应用BD方案治疗。

例3，男，58岁，主因“腹胀2个月余”入院。患者入院前1周就诊于当地医院，超声定位后抽取腹水3 000 ml，后为进一步诊断及治疗就诊于我院。入院查体：皮肤暗红，前胸、肩背部及双上臂可见多发大小不等毛细血管扩张。血常规：WBC 4.49×10^9^/L，RBC 8.86×10^12^/L，HGB 202 g/L，PLT 133×10^9^/L；生化常规：TP 68.9 g/L，GLO 35.5 g/L，Scr 115.6 µmol/L，EPO>758.00 mIU/ml；VEGF 77.19 pg/ml；血清IFE：在γ区可见一条单克隆IgG κ成分；血清游离κ链64.5 mg/L，游离λ链37.9 mg/L，κ/λ为1.7；血清M蛋白比例19.2％，血M蛋白定量13.23 g/L；血β_2_-MG 5.25 mg/L；尿IFE：在γ区可见一条单克隆轻链κ成分。骨髓细胞学检查：红系比例增高，骨髓及外周血中成熟红细胞堆积分布，不除外骨髓增殖性肿瘤。骨髓病理：少量限制性表达Kappa的浆细胞散在分布（<5％）。免疫表型：异常细胞群占有核细胞0.06％，强表达CD38、CD138、CD200，表达CD81，弱表达CD27、CD117、CD45、cKappa，不表达CD19、CD56、CD28、CD20、cLambda，结论符合异常浆细胞表型。腹部B超：腹盆腔积液，肝肾间包裹性积液伴分隔，肝实质回声稍粗。双肾实质回声增强，右肾周少量积液（最宽0.5 cm液性暗区）。肺血流灌注显像：静-动脉分流率约为7.6％。患者目前存在毛细血管扩张、EPO及HGB升高、肾周积液、M蛋白及异常浆细胞、肺内分流，诊断为TEMPI综合征，建议患者接受化疗，患者因经济原因暂拒绝治疗。

例4，男，44岁，主因“发现血红蛋白升高7年，乏力1个月”入院。患者2015年体检时查HGB 197 g/L，无头痛、眩晕、疲乏、呕血等不适，门诊诊断为PV，予羟基脲、阿司匹林治疗，近7年监测HGB波动于200～212 g/L，患者反复出现乏力、心悸，无胸闷、胸痛等不适，于2018年7月、2019年7月、2020年10月、2022年6月行红细胞单采术共4次。入院复查时查体：颜面潮红，双上肢、躯干可见红色毛细血管扩张。血常规：WBC 6.08×10^9^/L，RBC 4.55×10^12^/L，HGB 144 g/L，PLT 407×10^9^/L；生化常规：TP 59.8 g/L，白蛋白 39.0 g/L，GLO 20.8 g/L，Scr 96.8 µmol/L，钙2.24 mmol/L，LDH 165 U/L，铁蛋白27.9 ng/ml，EPO 139.19 mIU/ml；VEGF 138.04 pg/ml；未吸氧状态下血气分析：pH 7.39，碳酸氢盐23 mmol/L，二氧化碳分压37.9 mmHg，氧分压109 mmHg，氧饱和度98.6％，肺泡动脉氧分压差P（A-a）O_2_ 61 mmHg。血清IFE：在γ区可见一条单克隆IgG λ成分，血M蛋白定量5.9 g/L；血β_2_-MG 2.44 mg/L；尿IFE阴性。骨髓细胞学检查：骨髓增生活跃，粒、红、巨核系三系增生，红系增生明显，成熟红细胞排列密集，血小板成堆可见，NAP减少，浆细胞占1.0％。骨髓病理：造血细胞间见散在形态不典型浆细胞（占有核细胞<10％），结合临床及免疫组化标记结果，符合MGUS。免疫表型：异常浆细胞占0.06％，强表达CD56、CD138、CD38，表达CD27、cLambda，不表达CD19、CD81、CD117、CD13、CD33、CD22、CD20、CD28、cKappa。腹部脂肪活检特殊染色：刚果红（−/+）。P190、P210及JAK2 EXON12、JAK2/V617F基因突变均阴性。腹部B超：肝胆胰脾未见异常，双肾形态、大小正常。患者目前存在毛细血管扩张、EPO、HGB升高、M蛋白及异常浆细胞，考虑该患者符合TEMPI综合征诊断，建议其接受以硼替佐米为基础的诱导治疗，必要时行自体造血干细胞移植（auto-HSCT），患者表示暂不同意，后回当地未行特殊治疗。

## 讨论及文献复习

TEMPI综合征的概念在2011年被Sykes等[1]首次提出，2020年该学者首次提出TEMPI综合征的诊断标准[3]，将毛细血管扩张、EPO及HGB升高、单克隆丙种球蛋白病作为主要诊断，肾周积液和肺内分流作为次要诊断，此外还包括了静脉血栓形成等。目前文献仅报道20余例TEMPI综合征患者，年龄为40～70岁，男女比例相当，无明显的种族及地域差异，在报告的病例中，从出现症状到确诊的中位时间为10年（2个月～41年）[3]–[4]。

毛细血管扩张通常出现在患者的面部、上背部、胸部及上肢，下肢较少见。文献报道显示，除皮肤外，患者的毛细血管扩张还分布在结肠、颅骨和背侧椎体[5]–[6]，近期有文献报道，患者的慢性视网膜缺血伴微血管损伤可能与TEMPI综合征有关[7]。既往的病例报道对患者进行皮肤镜检查发现病变处为血管扩张和红色腔隙，组织病理学检查未见特异性变化[8]。很多患者的首发症状为红细胞增多及毛细血管扩张，由于红细胞增多而被误诊为PV并应用放血疗法治疗，但TEMPI综合征患者EPO升高、无JAK2基因突变，且骨髓形态学显示红系增生，未发现与骨髓增殖性肿瘤相关的特征[9]。单克隆丙种球蛋白病是TEMPI综合征的标志性特征，目前国内外报道病例的M蛋白以IgG κ型为主，部分患者为IgA型，有学者近期报道了1例IgM亚型的TEPMI综合征患者[1],[4],[10]–[12]。多数患者骨髓中浆细胞比例小于10％，无高钙血症、肾功能不全、贫血、骨病等临床表现，且血清游离轻链和血清游离轻链比值普遍正常或接近正常。目前肾周积液形成的机制尚不清楚，肾周积液穿刺结果显示成分与血浆相似，无病理性肿瘤细胞存在[5],[13]。当肾周积液达到一定水平时，可能会导致患者出现腹部饱胀感、腰痛、恶心、高血压或腹部肿块。肺内分流是TEMPI综合征的另一个表现，逐渐增加的分流指数可能导致低氧血症，发绀和劳力性呼吸困难是很多患者开始治疗的常见原因。还有一部分患者出现了血管疾病，如动静脉畸形、静脉血栓及自发性颅内出血[1]，本文例1也于近期出现了颅内出血，于当地医院治疗中，不除外与本病相关。另有文献报道了以难以愈合的双下肢皮肤溃疡为主要表现的TEMPI综合征患者，应用硼替佐米治疗后溃疡明显好转[14]。

目前TEMPI综合征的发病机制尚不明确，但对浆细胞的靶向治疗使患者病情获得了缓解，表明异常浆细胞克隆或单克隆丙种球蛋白病在疾病中发挥了重要甚至是核心作用。一些研究发现，单克隆分泌的细胞因子参与了疾病的发生发展。对患者的单克隆浆细胞进行全基因组测序发现了22q11.23的扩增，该位点对应的血清巨噬细胞迁移抑制因子（MIF）基因出现明显上调，且应用基于硼替佐米的方案治疗后患者血清MIF水平显著降低[15]。研究显示，MIF通过诱导VEGF和EPO表达在视网膜新生血管形成中起重要作用[16]，缺氧诱导因子（HIF 1）是EPO产生的关键转录因子，MIF可以诱导HIF 1的表达水平和转录活性上调[17]。另有研究显示，MIF可能在动静脉形成及肾周积液的产生中也发挥着重要作用[15],[18]，其发病机制还有待进一步探索。

随着对TEMPI综合征患者的研究，发现靶向浆细胞的治疗对患者有效。2011年TEMPI综合征被提出后，有学者应用蛋白酶体抑制剂硼替佐米治疗该疾病且患者获得了部分缓解甚至完全缓解[19]–[20]。若硼替佐米治疗后获得部分缓解，应用美法仑预处理序贯auto-HSCT可使患者获得长期完全缓解[21]。第一代免疫调节剂沙利度胺对该疾病患者尚无疗效[1]，但来那度胺可作为硼替佐米治疗后复发难治患者的挽救治疗方案[22]，近期的文献报道，应用泊马度胺序贯auto-HSCT作为二线治疗也取得了良好的疗效[23]。在CD38单抗达雷妥尤单抗被批准应用于治疗多发性骨髓瘤后，有学者尝试将其用于治疗复发/难治TEMPI综合征患者并获得了完全缓解[24]，但也有患者在疾病复发进展后应用达雷妥尤单抗无效并死于疾病进展[25]。由于目前对该疾病的了解程度有限，结合治疗的有效性和耐受性，Sykes等[3]建议应用达雷妥尤单抗或硼替佐米进行初始治疗，复发难治患者的治疗还有待进一步探索。
